# Unique Mitochondrial Single Nucleotide Polymorphisms Demonstrate Resolution Potential to Discriminate *Theileria parva* Vaccine and Buffalo-Derived Strains

**DOI:** 10.3390/life10120334

**Published:** 2020-12-08

**Authors:** Micky M. Mwamuye, Isaiah Obara, Khawla Elati, David Odongo, Mohammed A. Bakheit, Frans Jongejan, Ard M. Nijhof

**Affiliations:** 1 Institute for Parasitology and Tropical Veterinary Medicine, Freie Universität Berlin, Robert-von-Ostertag-Str. 7-13, 14163 Berlin, Germany; iobara@zedat.fu-berlin.de (I.O.); khawla.elati@fu-berlin.de (K.E.); 2 School of Biological Sciences, University of Nairobi, P.O. Box 30197-00100 Nairobi, Kenya; david.odongo@uonbi.ac.ke; 3 Department of Parasitology, Faculty of Veterinary Medicine, University of Khartoum, P.O. Box 321-11115 Khartoum, Sudan; mabakheit@uofk.edu; 4 Vectors and Vector-Borne Diseases Research Programme, Department of Veterinary Tropical Diseases, Faculty of Veterinary Science, University of Pretoria, Private Bag X04, 0110 Onderstepoort, South Africa; frans.jongejan@up.ac.za

**Keywords:** *Theileria parva*, mitogenomes, haplotypes, SNPs, live vaccine

## Abstract

Distinct pathogenic and epidemiological features underlie different *Theileria parva* strains resulting in different clinical manifestations of East Coast Fever and Corridor Disease in susceptible cattle. Unclear delineation of these strains limits the control of these diseases in endemic areas. Hence, an accurate characterization of strains can improve the treatment and prevention approaches as well as investigate their origin. Here, we describe a set of single nucleotide polymorphisms (SNPs) based on 13 near-complete mitogenomes of *T. parva* strains originating from East and Southern Africa, including the live vaccine stock strains. We identified 11 SNPs that are non-preferentially distributed within the coding and non-coding regions, all of which are synonymous except for two within the *cytochrome b* gene of buffalo-derived strains. Our analysis ascertains haplotype-specific mutations that segregate the different vaccine and the buffalo-derived strains except *T. parva-*Muguga and Serengeti-transformed strains suggesting a shared lineage between the latter two vaccine strains. Phylogenetic analyses including the mitogenomes of other *Theileria* species: *T. annulata*, *T. taurotragi*, and *T. lestoquardi*, with the latter two sequenced in this study for the first time, were congruent with nuclear-encoded genes. Importantly, we describe seven *T. parva* haplotypes characterized by synonymous SNPs and parsimony-informative characters with the other three transforming species mitogenomes. We anticipate that tracking *T. parva* mitochondrial haplotypes from this study will provide insight into the parasite’s epidemiological dynamics and underpin current control efforts.

## 1. Introduction

The protozoan parasite *Theileria parva* that causes East Coast fever (ECF) and Corridor Disease (CD) is considered among the most debilitating tick-borne pathogens in cattle over its endemic range in East, Central, and Southern Africa [1]. In typical ECF symptoms, the disease severity is mainly due to the parasites’ ability to transform host lymphocytes [2]. Parasitized lymphocytes proliferate uncontrollably and disseminate the dividing parasite into multiple host tissues. Their accumulation in the lungs triggers severe vasculitis, eventually resulting in respiratory failure with death occurring within three to four weeks of infection [3,4]. With mortalities of up to 100% in susceptible animals, an estimated one million die per year from an estimated risk population of 28 million cattle mainly belonging to livestock farmers with economically constrained livelihoods [5].

Thus, control of the parasite is urgent to livelihood improvement efforts among resource-poor farmers in sub-Saharan Africa, as highlighted by the World Organization for Animal Health (OIE) [6]. Current control methods include strict tick control measures to curtail pathogen transmission. However, this approach relies heavily on acaricide use, which is unsustainable in the long-run due to acaricide resistance challenges, and toxicity concerns in food and the environment [7]. Anti-theilerial chemotherapy is effective but only with early detection of the disease, which is impractical under field conditions [8,9].

Early observations that cattle acquire long-term immunity when challenged with infected ticks under a long-acting antibiotic treatment opened avenues for the development of an alternative control method based on live parasite stocks, which is called the infection and treatment method (ITM) [10,11]. ITM consists of inoculating cattle with cryopreserved *T. parva* sporozoites combined with simultaneous treatment with long-acting oxytetracyclines [12]. Early experiments revealed that there were varying cross-reactivities between geographical strains [13,14]. Due to this limitation, a cocktail of three immunizing parasite stocks known as the ‘Muguga cocktail’, comprising Serengeti-transformed, Kiambu 5, and Muguga strains, were combined to achieve broad protection against diverse field isolates [11]. Several other strains have been immunologically profiled to identify an isolate that cross-reacts to diverse field strains in ECF endemic areas. Among the identified strains was a Marikebuni stock isolated from the Kenyan Coast that showed cross-protection against several eastern African strains and, a Boleni strain from Zimbabwe which, apart from a cross-reactivity against Eastern and Central African strains, induced mild infections, hence eliminating the need for antibiotic use in ITM protocol [12].

Historically, ECF is traced to have originated from East Africa and spread southwards, first being reported in present-day Zimbabwe and eventually into South Africa [1]. Yet, it is notable that *T. parva* strains from different geographic regions have varying immunological profiles and epidemiological features. For example, an ability to induce a carrier state in which recovered animals remain infective to ticks has been demonstrated in some strains, enabling transmission between cattle by the vector tick, *Rhipicephalus appendiculatus* [15,16,17]. This persistence of vaccine strains raised initial concerns about spreading foreign parasite genotypes into endemic countries free of the vaccine parasite stocks, thereby possibly disrupting endemic stability [18].

By contrast, it is also known that some parasite strains, particularly of African buffalo (*Syncerus caffer*) origin, induce limited parasitosis and parasitemia, are non-persistent and not efficiently transmissible between cattle hosts [19]. These strains are known to cause a more acute clinical syndrome called Corridor Disease in areas where susceptible cattle are exposed to vector ticks infected on buffalo, which are the primary mammalian carrier hosts [20,21]. Based on its unique clinical presentation, which differs from classical ECF, these particular strains were initially recognized as *Theileria parva lawrencei* in earlier literature; however, this nomenclature was subsequently abolished with increasing molecular and antigenic data confirming similarities between the two strain populations [22,23,24]. Further, these data have revealed that cattle transmissible strains are a separately maintained subset population of those found in buffalo, and to differentiate between the two populations, *T. parva* strains are arbitrarily considered to be either of buffalo or cattle-derived for epidemiological reasons [25].

However, the genetic underpinnings of these strain differences are yet to be fully unraveled, and a precise delineation of the various genotypes is lacking [23]. This is partly because of the parasite’s biology, which renders it technically unamenable for genomic studies, especially in obtaining pure parasite DNA free from host-DNA contamination [26]. An accurate determination of the origin (buffalo or cattle derived) and geographic spread of strains will help intervention and control efforts. Additionally, accurate characterization of *T. parva* strains will help to track their frequency and distribution in specific populations, and to characterize breakthroughs in areas of live vaccine field deployments. Further, since *T. parva* has sexual reproductive phases that are associated with genetic recombination [27], unraveling the parasite genotypes could enhance the understanding of the long-term effects of live vaccine components in the field.

Owing to limited or no recombination, uniparental inheritance patterns and a high substitution rate relative to nuclear genomes, mitochondrial genome studies on related apicomplexan parasites have provided clues of the geographical origin and variants of parasites [28,29]. However, the utility of mitochondrial genomes in *T. parva* in delimiting the strains and their geographical origin remains unexplored. In this study, we sequenced the mitochondrial genomes of ten *T. parva* strains, found within the parasite’s currently known endemic range, as well as some characterized isolates used as vaccine strains. We also included the mitogenomes of nine other *T. parva* isolates assembled from their whole-genome data that are publicly available [30]. Further, this study assessed the divergence of *T. parva* from the closely related host-leukocyte transforming species *T. annulata*, *T. taurotragi* and *T. lestoquardi* with an aim to identify phylogenetically informative mitochondrial characters.

## 2. Materials and Methods

### 2.1. Source of Isolates

The parasite material for the different strains consisted of infected frozen ground-up tick supernatants (GUTS), salivary glands (SG), cattle whole blood, or infected lymphocyte cell lines (Table 1). The GUTS and SG were collected from archived *T. parva*-infected *R. appendiculatus* specimens from early live vaccination projects. Parasite DNA from GUTS, SG, and cell culture sample sources was extracted using the NucleoSpin Tissue kit (Macherey-Nagel, Düren, Germany), whereas the NucleoSpin Blood Mini (Macherey-Nagel) was used to extract DNA from blood samples.

### 2.2. Next-Generation Sequencing (NGS) T. parva Datasets

We additionally obtained publicly available whole-genome datasets of nine *T. parva* strains (DRR002439-46), downloaded in FASTQ from the NCBI (SRA accession number: DRA000613) for assembly of their mitogenomes sequences (Supplementary Table S2). The details of the parasite strains are described in a previous study [30]. All NGS datasets comprised 36 nucleotide, single-end sequence runs performed on the Illumina GAII Analyzer [30].

### 2.3. Mitogenome Amplification and Sequencing

Primers were designed based on an alignment of *T. parva* and *T. annulata* mitogenomes available in the GenBank (Accession nos. AB499089 and NW_001091933, respectively). A 5808 bp fragment was amplified from all isolated DNA extracts (0.5–5 ng) in 25 µL reaction volumes comprising; 0.5 U of S7 Fusion polymerase (Biozym Scientific, Hessisch Oldendorf, Germany), 5× GC Phusion buffer (ThermoFisher Scientific GmbH, Darmstadt, Germany), 200 mM of dNTPs mix, and 0.5 µM of each primer (Supplementary Table S1). The cycling conditions were as follows: 98 °C for 30 s, followed by 44 cycles of 98 °C for 15 s, 60 °C for 25 s, and 72 °C for 4 min. The final extension step was maintained at 72 °C for 10 min. Amplification of expected ~5.8 kb fragments was confirmed on 1.5% agarose gels stained with GRGreen DNA stain (Excellgene, Monthey, Switzerland) under UV trans-illumination. The amplicons were purified using GeneJET PCR Purification Kit (ThermoFisher Scientific GmbH, Darmstadt, Germany) before cloning using the Strataclone blunt vector (Agilent Technologies, USA) under the manufacturer’s instructions. The plasmid was purified using the GenUP™ Plasmid Kit (biotechrabbit GmbH, Berlin, Germany) and evaluated for targeted inserts based on the *Eco*RI digestion (ThermoFisher Scientific GmbH, Darmstadt, Germany). The plasmids were sequenced by Sanger technology using standard vector primers (LGC Genomics GmbH, Berlin, Germany) and 10 primers designed in this study to amplify overlapping regions of the mitogenome (Supplementary Table S1).

### 2.4. Assembly, Mapping, and Annotation

The Sanger generated sequences were assembled in Geneious prime 2020.2.3 (www.geneious.com) by creating consensus sequences from the approximately 1000 bp overlapping reads aligned to a reference mitogenome (GenBank Accession: AB499089), which is based on *T. parva* Muguga vaccine strain. Similarly, the Illumina NGS reads were mapped with reference to (AB499089) using the Geneious mapper under medium-low sensitivity with fine-tuning of at least five iterations. Consensus sequences were generated from contigs based on a threshold of at least 60% of the adjusted chromatogram quality of contributing bases, while ignoring reads mapped to multiple locations on the reference. The same GenBank reference was used to map and annotate protein-coding genes (PCGs) and the known rRNA genes based on nucleotide similarities.

### 2.5. Phylogenetic Analysis and Identification of Informative Single Nucleotide Polymorphisms (SNPs)

Mitogenome sequence alignments generated using MAFFT [40] as well as concatenated alignments of *cox1* and *cob* sequences with additional GenBank retrieved sequences of non-transforming *Theileria* spp. and *Babesia* spp. were used to infer maximum-likelihood phylogenies. We selected best-fit models for nucleotide substitution based on the lowest Bayesian information (BIC) scores calculated using the jModel Test 2 program and tested nodal support with 100 bootstrap replicates [41]. Phylogenetic trees were generated using PhyML implemented as a plugin within the Geneious software platform [42].

To avoid the challenges of missing data due to incomplete read coverage of the Illumina assemblies, we used only the Sanger data to generate the multiple alignments used for the SNPs detection. We aligned the ten *T. parva* Sanger-generated mitogenome sequences together with three other host-transforming species; *T. taurotragi*, *T. lestoquardi* and *T. annulata* (retrieved from GenBank: NW_001091933). SNPs were identified in Geneious prime with reference to the *T. parva* Muguga GenBank AB499089 sequence under default settings, with analysis of the variants on protein translations based on Mold-Protozoan Mitochondrial genetic code. We determined informative SNPs for the 13 mitogenomes under the parsimony optimality criterion with equal weights for all characters using PAUP*4.0 software [43].

### 2.6. T. parva Mitogenomes Haplotypes Definition and Network Analysis

Using a modified approach from [30], a second set of SNPs with consideration to the ten *T. parva* Sanger mitogenomes was extracted from the initial parsimony-informative SNPs. We used DnaSP v.6.12.03 on the second SNP data set to generate *T. parva* haplotype data [44] and a median-joining (M-J) network was constructed using Network V. 10 software (https://www.fluxus-technology.com/) under default settings to examine relationships among the *T. parva* mitogenomes [45]. Of the Illumina assembled mitogenomes, strains that had missing data with respect to the second SNP data set were excluded from the haplotype analysis.

## 3. Results

**Figure 1:**
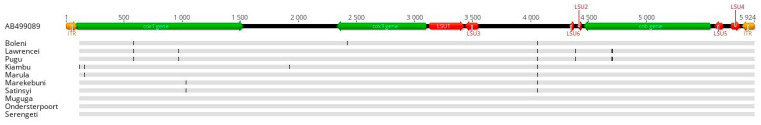
Linear map of *T. parva* mitochondrial genome and alignment showing the distribution of variants (SNPs) across the mitogenome sequences obtained by Sanger sequencing. cox1 and cox3: cytochrome oxidase subunits; cob: Cytochrome b; LSU: large subunit; ITR: Inverted terminal repeat region; SNPs: single nucleotide polymorphisms; vertical markings indicate polymorphisms in respective nucleotide sequence relative to the reference sequence AB499089 above.

At least ten bidirectional overlapping Sanger reads were obtained for each strain, which were assembled into mitogenomes sequences ranging in size from 5800 to 5811 bp. The sequences are archived in NCBI’s GenBank under accession numbers MW172707-MW172717; MW218514. The content and gene order for all ten *T. parva*, one *T. taurotragi*, and one *T. lestoquardi* mitogenomes were consistent with previous data, comprising three PCGs, fragmented rRNAs, and no tRNA [46] (Figure 1).

### 3.1. Divergence of T. parva from Other Host-Lymphocyte Transforming Theileria sp.

**Figure 2:**
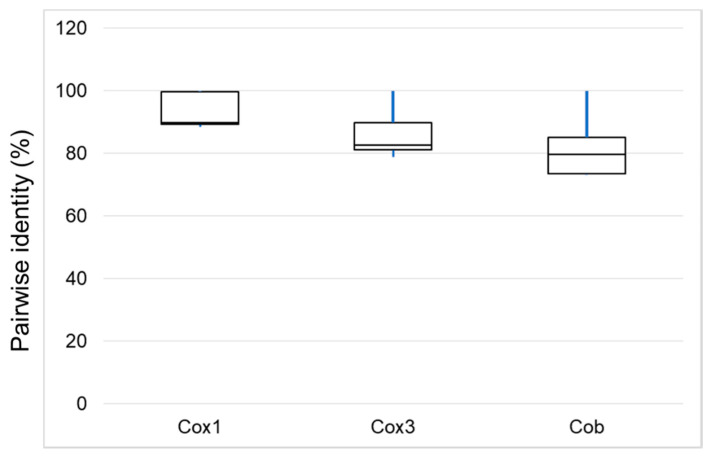
Percentage pairwise identity of the three protein-coding genes across the 14 mitogenomes analyzed in this study. The 25th and 75th percentiles are represented by the box limits; lines across the boxes indicate the median; whiskers extend to the maximum and minimum (%) identity values.

**Figure 3:**
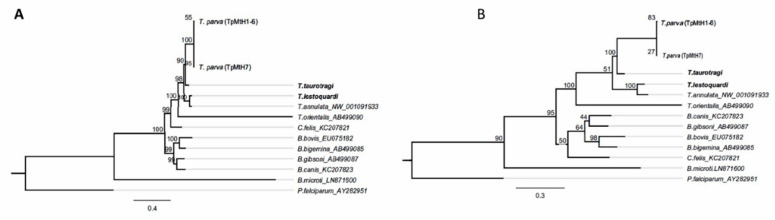
Maximum likelihood phylogeny based on (**A**) near-complete whole mitogenome sequences (~5.8 kb) and (**B**) cob sequences (~1.1 kb). The nucleotide substitution models for the tree constructions as determined by the lowest Bayesian information (BIC) values were TVM + G and GTR + G, respectively. Bootstrap values are based on 100 replicates. Sequences from this study are in bold.

**Figure A1:**
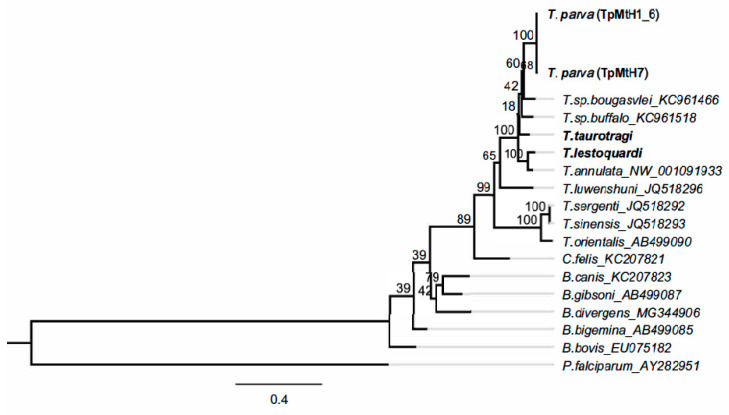
Maximum likelihood phylogeny based on *cox1* gene sequences (~1.4 kb). The nucleotide substitution model for the tree constructions as determined by the lowest BIC values was GTR + I + G. Bootstrap values are based on 100 replicates. Sequences from this study are in bold.

Due to length variations, we considered 5793 positions in the multiple alignment of the sanger-sequenced *T. parva* mitogenomes and the three additional host-lymphocyte transforming *Theileria* spp. Of the positions considered, there were 42 indels and 1036 SNPs. However, only 662 of the SNPs were parsimony informative across all mitogenome sequences. In terms of percentage identities of the PCG, cob was the most diverse gene, having 73.2–79.6% identity between *T. parva* Muguga strain and the three host-lymphocyte transforming *Theileria* (Figure 2). Phylogenetic analyses were congruent both using the whole mitogenomes and the concatenated gene sequences. In all instances, *T. annulata* and *T. lestoquardi* consistently formed an outgroup clade to *T. parva* and *T. taurotragi* (Figure 3; Appendix A, Figure A1).

### 3.2. T. parva Haplotype Analysis

**Figure 4:**
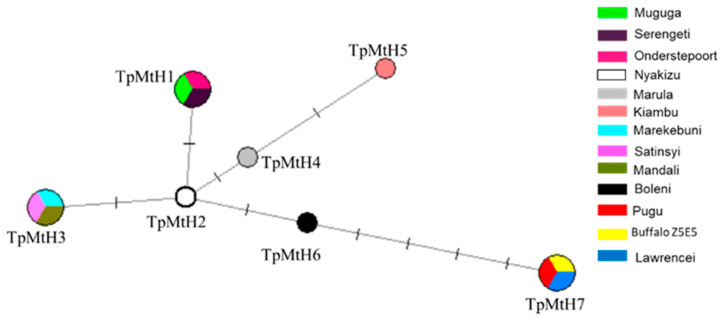
A median-joining (MJ) parsimony network for the 13 *T. parva* haplotype mitogenome sequences. Node labels TpMtH1-7 represents the unique haplotypes. Lines between nodes indicate mutation points. Larger and fractionated nodes indicate shared haplotypes with multiple strains, each marked with a different color key, as shown.

We used the extracted second set of SNPs, which comprised nine informative SNPs for *T. parva* haplotype analysis. As previously noted, we included three Illumina assembled mitogenomes (Nyakizu from Rwanda, MandaliZ22H10, and Buffalo Z5E5 from Zambia) that had data on all the determined nine informative SNPs, irrespective of the other missing regions lacking reads coverage. In total, 13 *T. parva* strains were considered for the haplotype analysis. The SNPs segregated the *T. parva* strains used into seven haplotypes, which we have identified in this study by assigning the TpmtH prefix numerically beginning with Muguga as a reference sequence (Figure 4). The Muguga strain isolate was assigned into one haplotype identical to Onderstepoort and Serengeti isolates (TpMtH1). Similarly, *T. parva lawrencei* (Manyara) isolated from an African buffalo, and Pugu I, both from Tanzania, together with a Zambian buffalo isolate (Buffalo Z5E5), formed one haplotype (TpMtH7) that was characterized by two SNPs within both the *cox* 1 and *cob* genes. We presumed Pugu I to have originated from buffalo *T. parva* based on analysis of its sporozoite surface (p67) antigen gene, which showed that it lacked the typical 129 bp deletion that is present in cattle transmissible *T. parva* [47,48]. The p67 sequence generated (See Appendix A for primer information and cycling conditions) for the Pugu I isolate is archived under accession no MW183674 in the GenBank.

**Figure 5:**
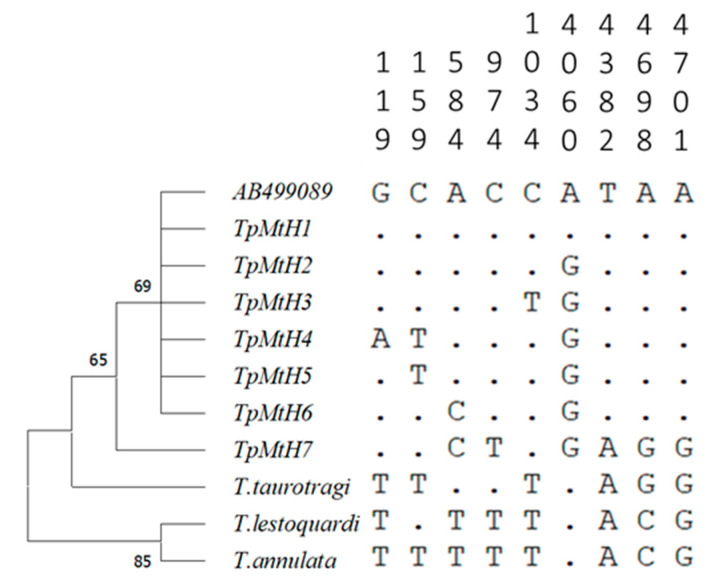
Phylogenetic grouping of the seven *T. parva* haplotypes identified in this study. The neighbor-joining tree is constructed based on the Jukes–Cantor Model using 9 *T. parva* only SNPs out of 662 informative SNPs extracted from 13-mitogenome sequences alignment that included; *T. taurotragi*, *T. annulata*, and *T. lestoquardi*. Numbers behind the nodes indicate bootstrap values based on 1000 replicates. The positions are relative to the AB499089 *T. parva* Muguga mitogenome sequence. Orthologous positions in the three other transforming *Theileria* are shown for comparison.

Further, the Marikebuni strain originating from the Kenyan coast, Mandali from Zambia, and Satinsyi strain from Rwanda formed one haplotype (TpMtH3) characterized by two SNP transitions relative to *T. parva* Muguga (Table 2; Figure 4). The Marula and Kiambu-V isolates formed independent haplotypes (TpMtH4 and 5), but differed with one SNP position (119) between them (Figure 5). The Boleni isolate (TpMtH6) possessed a transversion mutation within the *cox1* gene (SNP 584). This transversion mutation was also notable within the buffalo haplotype (TpMtH7). Interestingly, all haplotypes deviate from TpMtH1 by a transition (A→G) at SNP position 4060, which lies in a currently functionally unknown region, but appears to be ancestral in the other transforming *Theileria* (Table 2; Figure 5). This transition is the single defining SNP of the Nyakizu (Rwanda) strain from Muguga, and makes TmMtH2 a central node from which all other haplotypes deviate. However, there was no apparent differentiation by geographic origin as the M-J network nodes associated with multiple haplotypes clustered isolates of diverse origin (Figure 4).

### 3.3. Intraspecific Divergence among T. parva Strains

Relative to the GenBank reference AB499089, an alignment of 10 Sanger-sequenced *T. parva* mitogenomes showed variation at 11 sites, all of which were SNPs with no indels observed (Figure 1). Of the 11 SNPs, only three were found within the intergenic region and involved one transversion within the haplotype associated with buffalo *T. parva.* In total, there were three transversion SNPs positions, two of which were observed within the *cob* gene of the buffalo-associated haplotype. Among the genes, *cox3* was most conserved with only a single SNPs position within the Boleni mitogenome, while the *cox1* gene had five mutated positions, all of which were synonymous. The remaining two SNPs that were found within the *cob* gene sequences were non-synonymous and resulted in two contiguous amino acid substitutions in their predicted proteins (Table 2). Both substitutions involved the valine codon, which was replaced by an alanine amino acid codon. Notably, these substitutions were only in the haplotype associated with the buffalo *T. parva* isolates.

## 4. Discussion

In this study, we describe promising mitogenome-based SNPs that demonstrate precision and convenience in characterizing *T. parva* strains. Previously identified nuclear-based markers mainly based on a panel of mini- and micro-satellites are sometimes biased due to selective amplification of predominant strain clonotypes during passages through cattle and ticks [27,49]. In addition, since the design of the initial markers relied on the genome of *T. parva* Muguga stock, some markers are possibly biased in detecting diversity within this stock [50]. Although whole-genome SNPs analysis has been demonstrated to have high discriminatory power in typing vaccine strains, it is yet to find field applications [50,51]. Additionally, obtaining pure parasite DNA for whole-genome sequencing, especially for buffalo-derived *T. parva* is a hurdle due to its biology and may be complicated in the field where the parasite exists as a mixed diverse population [23,27].

Mitochondrial genomes and their individual genes have been extensively used to study phylogeny and applied in species identification and delimitation across broad taxonomic levels [52]. The majority of apicomplexan mitochondrial genomes that have been sequenced to date exhibit an extreme size reduction, containing at most three protein genes (*cox1*, *cox3*, and *cob*) and fragmented rRNA genes [53]. The extreme mitogenomes size reduction and a faster coalescence make mtDNA attractive to study differentiated *T. parva* population strains. Indeed, our analysis revealed haplotype-defining SNPs within the *T. parva* mitogenomes, which are parsimonious with other host-leukocytes transforming *Theileria* species. Based on median joining (MJ) parsimony analysis, the *T. parva* mtDNA sequences generated were segregated into an unambiguous network, congruent with the existence of multiple linked lineages. With respect to the *T. parva* Muguga isolate haplotype, each haplogroup was defined by synonymous nucleotide changes, except for non-synonymous changes leading to amino acid substitutions within the *cob* of the buffalo-associated strains; *T. parva lawrencei*, *T. parva* Buffalo Z5E5, and one field isolate, *T. parva* Pugu I.

Our analysis identifies *T. parva* Muguga and Serengeti-transformed as belonging to the same haplotype characterized by nine defining SNPs positions from the strains used in this study. This is not surprising as previous studies have demonstrated a similar monoclonal antibody profile and conservation on their known *T. parva* antigen coding genes [50,54]. Further, the two strains are strikingly similar at the whole genome level, with only 420 non-synonymous substitutions in Serengeti-transformed relative to the Muguga reference genome reported [50]. These substitutions occur in a paltry 53 genes (out of over 4000 *T. parva* genes) mainly within polymorphic multicopy gene families and ATP-binding cassette transporter genes located in subtelomeric ends [50]. With the almost similar identity of the two strains, our results, in addition to previous studies, question the necessity of both Muguga and Serengeti-transformed in the trivalent cocktail instead of a divalent cocktail containing either of the two and Kiambu-V. Interestingly, both Muguga and Serengeti-transformed *T. parva* strains also shared the same haplotype with a historical isolate *T. parva* Onderstepoort, a laboratory maintained stock isolated in 1937 on the farm Schoonspruit in the Transvaal, South Africa prior to ECF eradication in this country [1,37,55]. Earlier analyses on three *T. parva* antigen proteins; the Polymorphic immunodominant molecule (PIM), sporozoite surface protein (p67), and p104, have shown that these nuclear-encoded antigen genes are, in fact, identical to those of the Muguga parasite [50,56]. It is thus conceivable that the ECF-causing strains derive from a common lineage that can be inferred at the mitochondrial genome.

An important finding of this study is the clustering of buffalo-derived *T. parva* strains under one haplotype (TpMtH7) with the same nine SNPs. It is noteworthy that the buffalo strains used in this study originate from two different countries (Zambia and Tanzania), while the field isolate (Pugu I) was isolated during vaccine field trials in Tanzania. And although a Kenyan Buffalo (*T. parva* LAWR) from the NGS assembly was not included in the haplotype analysis due to missing data on SNP position (159), all its other SNPs positions also matched haplotype (TpMtH7) (data not shown). The buffalo has long been recognized as the natural reservoir of *T. parva*. The *T. parva* strains maintained in cattle are considered a subset population from that maintained in buffalo [23,25]. However, there has not been a definitive genetic basis to differentiate what constitutes a buffalo-derived *T. parva* and a cattle-derived *T. parva* or whether their designation as a single species is justified [23,56]. The available approach of their differentiation based on the p67 alleles only provides a preliminary indication of presumptive exposure of cattle to buffalo *T. parva* based on alleles-2, 3, and 4, which are considered highly probable to be of buffalo origin in contrast to allele-1 that is found in cattle transmitted *T. parva,* but does not necessarily preclude its presence in buffalo [48,49,57]. Our analysis suggests strain defining mitochondrial SNPs that are potential markers for buffalo-derived *T. parva* lineages.

Noticeably, the Boleni strain formed a separate haplotype (TpMt6) that shared a transversion mutation within the *coxI* gene with the buffalo haplotype. This strain was isolated from Zimbabwe from a farm that had experienced a severe theileriosis outbreak in January 1978 [31]. Under the now obsolete trinomial nomenclature of *T. parva*, it was named *Theileria parva bovis*, which was associated to what was referred to as January disease [58]. The delineation of this strain from our data is thus a significant find as it agrees with the epidemiological distinctions that have been apparent from earlier investigations on theilerioses caused by *T. parva*. Further, our analysis identifies the Kenyan-Marekebuni, Zambian-Mandali, and Rwandese Satinsyi strains as one haplotype (TpMtH3). Although the shared haplotypes from widely separated regions may suggest a lack of geographical differentiation of the haplotypes, our observations could also be because of a limited sample size as well as through spread by carrier animals.

A high level of interspecies divergence among the transforming *Theileria* is observed that is characterized by up to 42 indels with respect to *T. parva* Muguga. However, a limited polymorphism is observed amongst the 13 *T. parva* mitogenomes analyzed, which is also observed in other apicomplexan species such as *Plasmodium falciparum* [59]. Of the eleven *T. parva* SNPs observed, only nine were informative. We modestly suppose this may be convenient compared to whole-genome-based SNPs in which up to >120,000 SNPs have been observed in buffalo strains alone [30]. Additionally, we think our approach to defining SNPs that are foremost parsimonious with other leukocyte transforming *Theileria* provides initial indications on the potential of the identified SNPs to be informative for typing of recently diverged field *T. parva* strains from common leukocyte transforming ancestor. Nonetheless, further investigation to test the utility of the SNPs is necessary with a larger field population across the *T. parva* endemic range, especially in wildlife-livestock areas where ‘breakthrough’ infections against the trivalent live vaccine are known to occur.

The phylogenetic analysis of both the full-length near-complete mitochondrial genomes and the concatenated *cox1* and *cob* genes place *T. parva* and *T. taurotragi* in one clade, consistent with previous analyses using nuclear genes such as the 18S RNA gene. The same phylogenetic tree topology is maintained with the sporozoite surface protein gene and its orthologues in respective leukocyte host-transforming species [60]. Thus, the mitogenomes data’s observed congruency with nuclear-based data rules out possibilities of inheritance patterns specific to mitochondria in our analysis.

Our data indicate *T. annulata* and *T. lestoquardi* form an outgroup clade among the transforming parasites, reflecting an allopatric speciation separation from *T. parva* and *T. taurotragi,* and conforms to their currently known demography. Noticeably, *T. taurotragi* was initially described as a parasite of the eland (*Taurotragus oryx*) [61], but is also reported to cause infections in cattle in the known endemic range (Eastern, Central, and Southern Africa) of *T. parva* and its tick vector *R. appendiculatus*, alongside other tick vectors [38]. As such, co-infections of *T. taurotragi* and *T. parva* are, in fact, frequently common [62]. While the pathogenicity of *T. taurotragi* in cattle is not clearly understood, it has been shown to transform a wide range of host cells in in vitro studies [63], and has been associated with cases of cerebral theileriosis (BCT) [38,64].

Similarly sympatric, *T. annulata* and *T. lestoquardi*, occur within the same currently known endemic range (N. Africa, S. Asia, and S. Europe) and are transmitted by ticks belonging to *Hyalomma* genus. Both are important parasites responsible for heavy economic losses and have an intertwined epidemiology that poses interpretation challenges in their overlap in affected countries [65]. *Theileria lestoquardi* is a parasite of small ungulates and causes malignant ovine theileriosis, while *T. annulata* causes bovine tropical theileriosis but also co-infects with the former in sheep [65,66,67].

In conclusion, this study catalogs SNPs based on mitogenomes of characterized *T. parva* strains and vaccine stocks that can facilitate their tracking in the field. We identify haplotypes defined by SNPs that are initially parsimonious among transforming *Theileria*; *T. parva*, *T. annulata*, *T. taurotragi*, and *T. lestoquardi* mitogenomes, the latter two reported herein for the first time. We anticipate that the knowledge of the circulating haplotypes with reference to the live vaccine strains haplotypes will be insightful in characterizing *T. parva* epidemiology with important implications for control, and have a predictive value on the success of live vaccine deployments besides characterization of breakthrough infections.

## Figures and Tables

**Table 1:** Parasite material and origin of isolates used in the current study.

Strain/Isolate	Origin	Material Used	Year Created *	Reference
*T. parva* (Serengeti-transformed)	Tanzania	GUTS	1981	[31]
*T. parva* (Boleni)	Zimbabwe	GUTS	1980	[32]
*T. parva* (Pugu I)	Tanzania	Cell culture	1977	n.a.
*T. parva* lawrencei (Manyara)	Tanzania	GUTS	1980	[33]
*T. parva* (Satinsyi)	Rwanda	GUTS	1981	n.a.
*T. parva* (Kiambu)	Kenya	Salivary glands	1980	[34]
*T. parva* (Marikebuni)	Kenya	GUTS	1985	[35]
*T. parva* (Marula)	Kenya	Blood	2000	[20]
*T. parva* (Muguga)	Kenya	Salivary glands	1991	[36]
*T. parva* (Onderstepoort)	South Africa	GUTS	1988	[37]
*T. taurotragi*	Tanzania	Blood	2003	[38]
*T. lestoquardi* (Atbara)	Sudan	Cell culture	2001	[39]

* Indicates the year the material used for this study was created, which may differ from the time when the parasite was first isolated for some strains. n.a.: not available.

**Table 2:** SNPs among the seven haplotypes based on the GenBank reference AB499089. The reference sequence matches haplotype TpMtH1 in the present study.

Haplotype	Gene	Variant Type	Change	Codon Change	Codon Position	AA Change	Protein Effect
	*Cox1*						
TpMtH3		Transition	C→T	GCC→GCT	951		
TpMtH4		Transition	C→T	CTG→TTG	76		
TpMtH5		Transition	G→A	GTG→GTA	36		
		Transition	C→T	CTG→TTG	76		
TpMtH6		Transversion	A→C	GTA→GTC	501		
TpMtH7		Transversion	A→C	GTA→GTC	501		
		Transition	C→T	TAC→TAT	891		
	*Cox3*						
TpMtH6		Transition	T→C	CAA→CAG	555		
	*Cob*						
TpMtH7		Transition	A→G	GTT→GCT	848	V→A	Substitution
		Transition	A→G	GTA→GCA	851	V→A	Substitution
	Intergenic				**SNP position**		
TpMtH2		Transition	A→G		4060		
TpMtH3		Transition	A→G		4060		
TpMtH4		Transition	A→G		4060		
TpMtH5		Transition	T→C		1924		
		Transition	A→G		4060		
TpMtH6		Transition	A→G		4060		
TpMtH7		Transition	A→G		4060		
		Transversion	T→A		4382		

## Supplementary Materials

The following are available online at https://www.mdpi.com/2075-1729/10/12/334/s1. Table S1: Amplification and sequencing primer sequences, Table S2: Summary of NGS reads (SRA accession number: DRA000613) mapped to *T. parva* Muguga-mitochondrial sequence (AB499089).

## Appendix A

The pugu *p67* gene was amplified by PCR in a 25 μL reaction using published primers [48]. Briefly, the amplification reaction contained; 5× High-Fidelity (HF) Phusion buffer (ThermoFisher Scientific), 200 μM dNTPs (Biozym Scientific GmbH), 0.5 μM forward primer (IL 6133: 5′-ACAAACACAATCCCAAGTTC-3′), 0.5 μM reverse primers; IL 7922: 5′-CCTTTACTACGTTGGCG-3′), 0.02 U/μL HF Phusion DNA polymerase, sterile PCR-grade water (Carl Roth GmbH, Karlsruhe), and 2 μL of template DNA. The amplification cycle was set as follows; 98 °C for 30 s, followed by 35 cycles of 98 °C for 30 s, an annealing step at 58 °C for 40 s, and elongation at 72 °C for 1 min. The final extension step was 10 min at 72 °C. The p67 amplicon was purified using GeneJET PCR Purification Kit (ThermoFisher Scientific), and sequenced using Sanger technology (LGC Genomics, Berlin). The obtained sequence was translated and evaluated for the presence/absence of the 43 amino-acid deletions used to categorize cattle and buffalo derived p67 sequences.
