# Supplementary material for: Unique Mitochondrial Single Nucleotide Polymorphisms Demonstrate Resolution Potential to Discriminate Theileria parva Vaccine and Buffalo-Derived Strains
Source: Life (Basel). 2020 Dec 8;10(12):334. doi: 10.3390/life10120334 (PMC7764068; doi:10.3390/life10120334)
Supplement: Supplementary file 1 [file life-10-00334-s001.pdf]

life

Supplementary file

Unique Mitochondrial Single Nucleotide Polymorphisms demonstrate resolution potential to discriminate *Theileria parva* Vaccine and Buffalo-Derived Strains

Micky M. Mwamuye [1,*], Isaiah Obara [1], Khawla Elati [1], David Odongo [2], Mohammed A. Bakheit [3], Frans Jongejan [4] and Ard M. Nijhof [1,*]

[1] Institute for Parasitology and Tropical Veterinary Medicine, Freie Universität Berlin, 13, 14163 Berlin, Germany; iobara@zedat.fu-berlin.de (I.O.); khawla.elati@fu-berlin.de (K.E.)
[2] School of Biological Sciences, University of Nairobi, P.O. Box 30197–00100, Nairobi, Kenya; david.odongo@uonbi.ac.ke
[3] Department of Parasitology, Faculty of Veterinary Medicine, University of Khartoum, P.O. Box 321–11115 Khartoum, Sudan; mabakheit@uofk.edu
[4] Vectors and Vector-borne Diseases Research Programme, Department of Veterinary Tropical Diseases, Faculty of Veterinary Science, University of Pretoria, Private Bag X04, Onderstepoort 0110, South Africa; frans.jongejan@up.ac.za
* Correspondence: micky.mwamuye@fu-berlin.de (M.M.M.); ard.nijhof@fu-berlin.de (A.M.N.); Tel.: +49-30-838-62326 (A.M.N.)

Supplementary Materials

Table S1. Amplification and sequencing primer sequences.

Reaction.	Primer name	Sequence
Amplification	Tp_mit_F79	5'-GTGTCAGGAAATCATAAAATTATTGG-'3
	Tp_mit_R5885	5'-TGAGTAAGAATAATGATACTCAAATATATGTCG-'3
Sanger sequencing		
	Tp_mit_F118	5'-GTGGCTGGCTTATTGGTTCG-'3
	Tp_mit_R965	5'-GCGAGTATCTGCTTCCAAACC-'3
	Tp_mit_F905	5'-GTTAGGTTGTTTGGTTTGGGGAC-'3
	Tp_mit_R5610	5'-TTTAGTGAAGGAACTTGACAGGTACA -'3
	Tp_mit_R2080	5'-TTTGAACACACTGCTCGACAC-'3
	Tp_mit_R1379	5'-CAGGATAATCTGGTATTCTTCTTGG-'3
	Tp_mit_F1863	5'-CAAGGTAGTTGACAGTGAACTTGTAGC-'3
	Tp_mit_F3056	5'-CGCTGTTTCGCATTTGACTAC-'3
	Tp_mit_R4186	5'-TTCTTTGCCTTGGATGTCAGTTAG-'3
	Tp_mit_F3659	5'-CAATCCTTATGTATGCTTGAATGCTG-'3

M13-24F- Blue*	5'-GTAAAACGACGGCCAGTGAGCGCG-'3
M13-24R- Blue*	5'-AACAGCTATGACCATGATTACGCC-'3

* pSC – B – amp/kan PCR cloning vector standard sequencing primers.

Table S2. Summary of NGS reads (SRA accession number: DRA000613) mapped to *T. parva* muguga- mitochondrial sequence (AB499089).

Strain	Accession no.	Origin	Total reads downloaded	Mapped reads	Read coverage
Buffalo Z5E5	DRR002446	Zambia	14,821,055	2,627	96.4%
Nyakizu	DRR002443	Rwanda	31,095,446	2,574	98.9%
Buffalo LAWR	DRR002445	Kenya	17,072,361	2,021	95.0%
Entebbe	DRR002442	Uganda	10,171,313	1,826	96.8%
ChitongoZ2	DRR002438	Zambia	14,405,286	1,198	94.4%
MandaliZ22H10	DRR002441	Zambia	16,362,288	591	91.1%
Katumba	DRR002444	Tanzania	35,406,726	463	85.3%
KiambuZ464/C12	DRR002440	Kenya	15,848,448	428	84.1%
KateteB2	DRR002439	Zambia	16,558,766	426	81.1%
